# Les résultats de la technique de Latarjet dans l´instabilité antérieure de l´épaule en fonction de la position radiologique de la butée coracoïdienne

**DOI:** 10.11604/pamj.2021.38.215.21339

**Published:** 2021-02-24

**Authors:** Oueslati Achraf, Rafrafi Abderrazzek, Znagui Talel, Saadi Saber, Nouisri Lotfi

**Affiliations:** 1 Service d´Orthopédie et de Traumatologie, Hôpital Militaire Principale d´Instruction de Tunis, Montfleury, Tunis, Tunisie

**Keywords:** Épaule, instabilité antérieure, technique de Latarjet, Shoulder, anterior instability, Latarjet technique

## Abstract

L´instabilité antérieure de l´épaule suite à une luxation traumatique chez l´adulte jeune est une complication fréquente. La procédure de Latarjet est la technique la plus utilisée pour traiter cette instabilité consiste à réaliser une butée coracoïdienne au niveau du bord antérieur de la glène. Cependant, l´exposition articulaire au cours de cette technique est souvent limitée et rend le positionnement de la butée difficile. Pour évaluer l´effet de la position de la butée coracoïdienne sur la qualité du résultat fonctionnelle à court et moyen termes. Nous avons étudié chez 70 patients la position de la butée coracoïdienne sur la radiographie standard post-opératoire et le résultat fonctionnel par le score du Duplay et le taux de satisfaction. L´âge moyen était de 25 ans et demi, le sex-ratio était de neuf et le recul moyen était de 6,5 ans. L´étude radiologique a montré que la butée coracoïdienne était en position sus équatoriale ou non affleurante du rebord glénoïdien antérieur (trop interne ou saillante en intra-articulaire) dans 20% des cas. Ce groupe a présenté une baisse du score moyen de stabilité par 7,68 points, de la douleur par 10,04 points et du score global de Duplay par 13,3 points, ainsi qu´une augmentation significative du taux d´arthrose gléno-humérale. Ainsi, une butée coracoïdienne en position sus équatoriale ou non affleurante a un effet délétère sur la qualité des résultats fonctionnels de la technique de Latarjet.

## Abstract (en)

Anterior shoulder instability following traumatic dislocation in young adults is a common complication. The Latarjet procedure is the most widely used technique for treating this instability. It is based on coracoid abutment repair of the anterior edge of the glenoid. However, joint exposure during this surgical technique is often limited and makes it difficult to position the abutment. The purpose of this study was to evaluate the result of coracoid abutment procedure on functional outcome in the short and medium term. We assessed the positioning of the coracoid abutment on standard postoperative X-rays and the functional outcome according to Duplay score as well as the satisfaction rate in 70 patients. The average age of patients was 25 and a half years, the sex-ratio was nine, and the mean follow-up period was 6.5 years. Radiological examination showed that coracoid abutment was in subequatorial or non-flush position with respect to the anterior glenoid rim (too internal or protruding in intra-articular position) in 20% of cases. This group showed a drop in mean stability score by 7.68 points, in pain by 10.04 points and in Duplay’s overall score by 13.3 points as well as a significant increase in the level of glenohumeral arthrosis. This study highlights that coracoid abutment in subequatorial or non-flush position has deleterious effect on the functional outcome of the Latarjet procedure.

## Introduction

L'instabilité antérieure de l´épaule constitue une des complications les plus importantes de la luxation antéro-interne post traumatique de l´épaule. Cette complication constitue un handicap fonctionnel majeur surtout chez des sujets jeunes et actifs. Parmi les méthodes chirurgicales pour la traiter, l´opération de Latarjet [1] constitue la technique la plus répandue vu son efficacité et son faible taux de récidive. Cette technique consiste à mobiliser l´apophyse coracoïde au niveau du bord antérieur de la glène a fin d´assuré la stabilité de l´épaule. Cependant, l´exposition articulaire au cours de cette technique est souvent limitée et rend le positionnement du greffon difficile, même pour les équipes les plus expérimentées. Le but de notre étude a été d´évaluer chez 70 cas d´instabilité antérieure de l´épaule, traités par la technique de Latarjet au service d´orthopédie-traumatologie de l´hôpital militaire de Tunis, la position de la butée coracoïdienne sur des clichés de radiographies standards post opératoire, et d´analyser l´effet de cette position sur la qualité du résultat fonctionnelle.

## Méthodes

Il s´agit d´une étude rétrospective, mono centrique, descriptive et portant sur des patients ayant été hospitalisés pour une instabilité antérieure chronique de l'épaule post traumatique, traitée par l´intervention de Latarjet au service d´orthopédie-traumatologie de l´hôpital militaire de Tunis, sur une période de 10 ans entre 2004 et 2014. Nous avons inclus dans cette étude les patients ayant eu des luxations antéro-internes récidivantes de l'épaule documentée, traitée uniquement selon la technique de Latarjet avec un recul supérieur à un an. Les autres types d'instabilités de l'épaule et les instabilités traitées par d´autres interventions n´ont pas été inclus. Et nous avons exclu tous les patients dont les dossiers médicaux étaient inexploitables et les patients injoignables ou refusant de répondre à la convocation. Soixante-dix dossiers ont rempli les critères précités. Tous les patients ont été opérés par des chirurgiens seniors. La voie d´abord était la voie delto-pectorale. L´attitude des opérateurs vis à vis au muscle subscapulaire a été une simple décision dans tous les cas. Les lésions du bourrelet et les fragments ostéochondraux ont été réséqués. Un avivement de la butée et de la glène a été réalisé dans tous les cas. La butée coracoïdienne a été positionnée en position debout dans 65 cas et en position couchée dans 5 cas. La fixation de la butée a été réalisée par une seule vis AO 4.5 corticale dans 67 cas et par deux vis 3.5 corticales dans 3 cas. Tous les patients ont reçu un traitement antalgique post-opératoire par un antalgique palier 1 et une immobilisation initiale puis une rééducation progressive.

**Figure 1:**
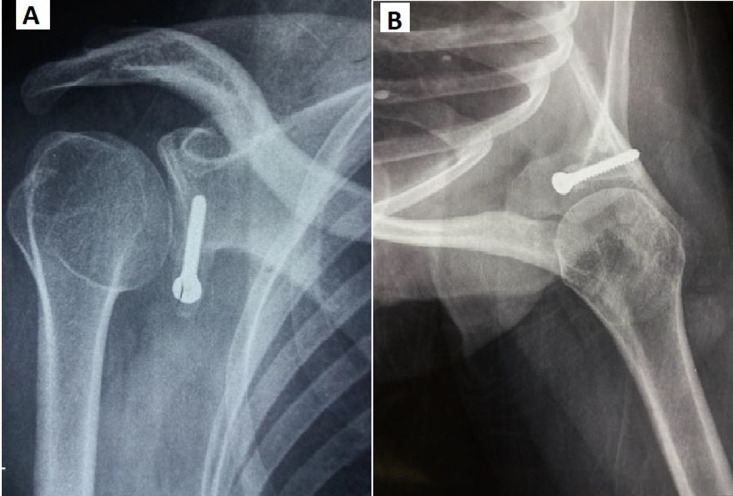
radiographie épaule post-opératoire; a) incidence de face rotation neutre; b) incidence profile de Bernageau

L´évaluation clinique a été effectuée de façon objective selon le score de Walch-Duplay [2], et de façon subjective par des questions: êtes-vous satisfaits, déçus, mécontents? Les résultats radiologiques ont été analysés sur un cliché de face et un cliché de profil de Bernageau [3] (Figure 1). Sur ces clichés nous avons étudié: la position de la butée coracoïdienne par apport à l'équateur de la glène et par rapport à la ligne de condensation correspondant au rebord glénoïdien antérieur. Nous avons cherché également, l´existence ou non d´une fracture, d´une lyse et d´une pseudarthrose de la butée et l´existence ou non d´une arthrose gléno-humérale classée selon la classification de Samilson et Prieto [4]. Nous avons utilisé le logiciel SPSS pour notre étude statistique. Pour l´étude descriptive nous avons calculé des fréquences simples et des fréquences relatives (pourcentage) pour des variables qualitatives et des moyennes et les écarts types pour les variables quantitatives. Pour l´étude analytique les comparaisons de deux variables qualitatives ont été effectuées par le test chi-deux de Pearson et en cas de non-validité de ce test, nous avons utilisé le test de Fischer. Les comparaisons entre une variable qualitative binaire et une variable quantitative ont été effectuées avec le test t de Student. Le seuil de significativité p a été fixé à 5%.

## Résultats

L´âge moyen au moment de la chirurgie était de 25 ans et demi (18 - 41 ans). Le recul moyen était de 6.5 ans. Notre série a comporté 63 hommes et 7 femmes, soit un sex-ratio de 9. La chirurgie a intéressé le côté dominant dans 63% des cas. Sept patients étaient des sportifs de compétition, 39 patients étaient des sportifs de loisir et les restes étaient soit des sportives occasionnelles ou sédentaires. L´étude des résultats cliniques selon le score de Duplay est présentée par le Tableau 1. Pour l'évaluation subjective: 62 patients (89%) déclaraient être satisfaits, 5 patients (7%) étaient déçus et 3 patients (4%) étaient mécontents. L´analyse radiologique objective une butée en position sous équatoriale dans 68 cas (97%). Par rapport au rebord antérieur de la glène, la butée était en position affleurante du rebord glénoïdien antérieur dans 56 cas (80%), débordante intra articulaire dans 12 cas (17%), et trop interne par rapport au rebord glénoïdien dans 2 cas (3%). Nous avons eu 10 cas de lyse de la butée, un seul cas de pseudarthrose et aucun cas de fractures de la butée. La principale complication était l´arthrose gléno-humérale. Onze patients (16%) ont développé une arthrose gléno-humérale post-opératoires; parmi ces patients, 9 cas (12.85%) était de stade 1 et deux cas (2.85%) était de stade 2 selon la classification de Samilson. L´étude de l´association entre la position de la butée coracoïdienne et les valeurs moyennes des scores de la stabilité, la douleur et le score global de Duplay sont présentés par le Tableau 2. L´association entre la position de la butée et le taux d´arthrose post opératoire est également présentée par le Tableau 2.

**Tableau 1:** les résultats cliniques selon le score de Duplay et ses quatre paramètres

Paramètres		Effectif	Pourcentage	Le score moyen
A	0 points	2	3%	21,15 points
	+10 points	7	10%	
	+15 points	11	16%	
	+25 points	50	71%	
B	-25points	1	1%	22,5 points
	0 points	2	3%	
	+15points	12	17%	
	+25 points	55	79%	
C	0 points	8	11%	19,35 points
	+15 points	20	29%	
	+25 points	42	60%	
D	0 points	1	1%	23,6 points
	+5 points	6	9%	
	+15 points	24	34%	
	+25 points	39	56%	
A+B+C+D				86,6 points

A - activités de la vie courante, B - stabilité, C - douleur, D - mobilité globale, A+B+C+D - score global de Duplay.

**Tableau 2:** étude des associations statistiques entre la position de la butée coracoïdienne et le score moyen de la stabilité, la douleur, le score global de Duplay et le risque d´arthrose post opératoire

		Butée affleurante et sous équatoriale	Autres	p - value
**Stabilité**	-25 points	0	1	<0.0001
	0 points	0	2	
	+15 points	9	3	
	+25 points	47	8	
	**Score moyen**	23,39	15,71	
**Douleur**	0 points	2	6	0.004
	+15 points	15	5	
	+25 points	39	3	
	**Score moyen**	21,51	11,47	
**Score de Duplay (score moyen)**		89,19	76,29	0.0001
**L'arthrose post opératoire**	Stade 1	2	7	0.001
	Stade 2	0	2	
	%	3%	64%	

## Discussion

**Figure 2:**
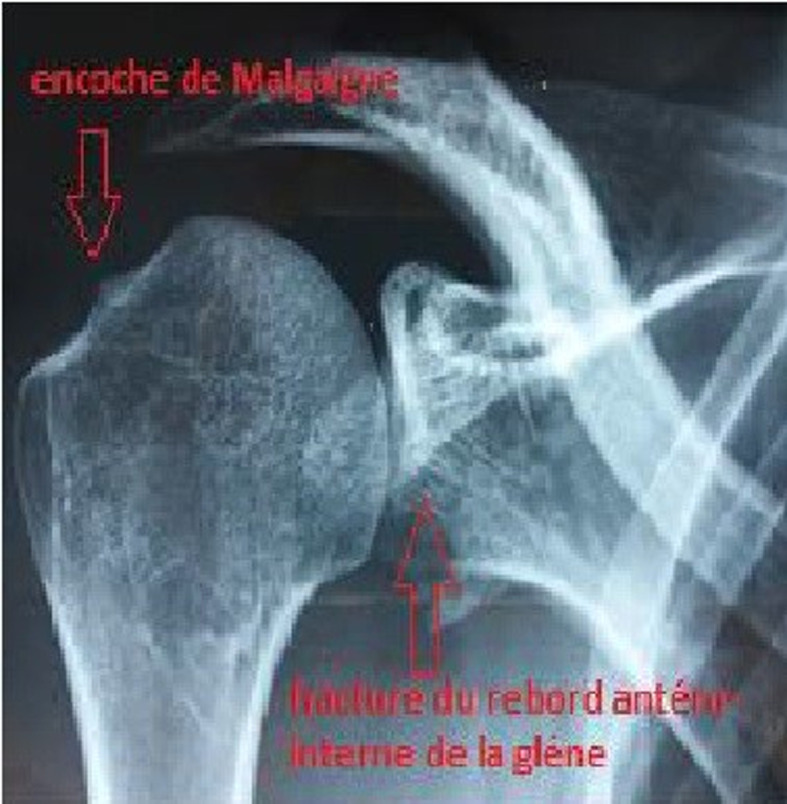
radiographie épaule face en rotation neutre montrant une fracture de rebord antérieur de la glène avec encoche de Malgaigne

La technique de Latarjet était efficace dans le traitement de l´instabilité antérieure de l´épaule avec un bon résultat fonctionnel confirmé par le score de Duplay et taux important de satisfaction des patients. Cependant, 20% des patients ont eu une butée coracoïdienne en position sus équatoriale, ou non affleurante du rebord glénoïdien antérieur (trop interne ou débordante en intra-articulaire). Ces patients ont exprimé davantage des sensations d´instabilité et des douleurs, et une diminution statiquement significative du score de Duplay. Nous avons observé également un taux d´arthrose post opératoire plus important chez ses patients. Quoique notre étude ait comporté certains biais. Essentiellement, un biais de sélection relatif au caractère rétrospectif de l´étude et un recul moyen de 6.5 ans relativement court, comparé aux autres séries. Notre étude se distingue par le caractère homogène de la population, la majorité des patients étaient des militaires assez jeunes et disposaient d´une bonne condition physique. De plus nos critères d´exclusion stricte ont éliminé tous les dossiers inexploitables. Comme notre série, la plupart des séries qui ont étudié l´instabilité antérieure de l´épaule ont rapporté une population jeune de sexe masculin [5,6], car avec l´âge il y a une perte d´élasticité capsulo-ligamentaire et une diminution de la densité osseuse, on a tendance donc à avoir plus de fractures de l´humérus que de luxations. En effet, au cours des épisodes de luxations, il se produit des lésions de passage. Ces lésions peuvent atteindre les éléments capsulo-labrale et les structures osseuses, essentiellement l´encoche de Malgaigne au niveau de la tête humérale et un écoulement ou fracture du rebord antérieur de la glène (Figure 2), diminuant ainsi la surface articulaire. Le transfert de l´apophyse coracoïde a pour objectif de former une butée pour augmenter la surface articulaire de la glène. Pour Goutallier et Glorion [7], cet agrandissement de la glène permet d'éloigner l'encoche de Malgaigne du rebord antérieur de la glène, évitant ainsi les récidives de luxation par effet came. De plus ce transfert permet le déplacement du tendon conjoint pour jouer le rôle d´un stabilisateur antérieur de l´articulation gléno-humérale par l'effet hamac décrit par Patte *et al*. [8].

**Figure 3:**
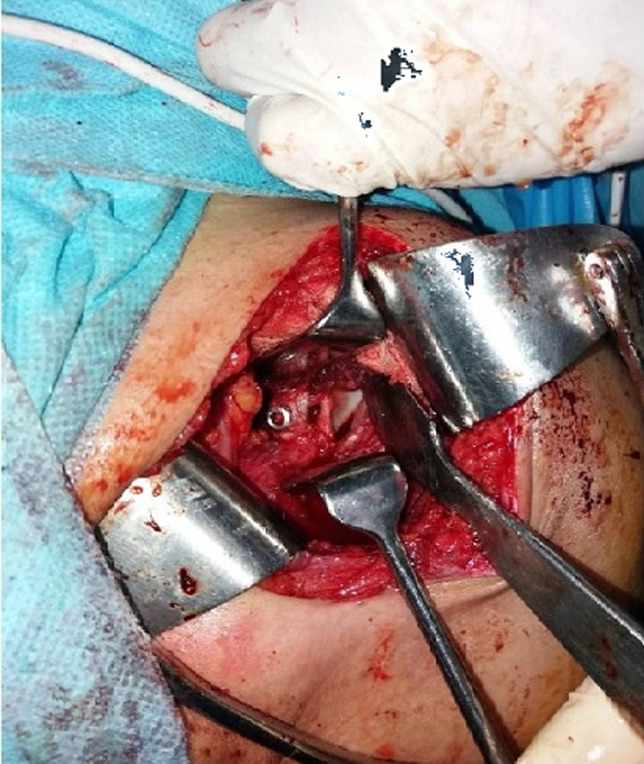
photo per-opératoire d´une fixation de la butée couchée en position affleurante et par une seule vis AO 4,5mm

Ainsi, une position sus équatoriale de la butée [9,10] ou une butée trop interne sont à l'origine de récidive d'instabilité post-opératoire [11], alors qu´une butée débordante entraine un conflit tête-coracoïde [12]. Et ces deux situations sont source des douleurs [9,12]. Cela est en concordance avec nos résultats qui ont objectivé une diminution du score moyen de la stabilité, de la douleur et du score global de Duplay chez le groupe des patients avec une butée sus équatoriale ou non affleurante. Malgré la prise en charge par une équipe qui dispose d´une bonne expérience, le pourcentage des patients qui appartenaient à ce groupe a atteint 20% dans notre série. Ceci a été comparable au taux rapporter par la littérature. Selon la série de Hovelius *et al*. [13], ce taux est de 36% et il atteint 45% selon la série de Huguet *et al*. [12]. Ces taux élevés sont expliqués par l'exposition réduite de l'articulation gléno-humérale lors d´un abord delto-pectorale avec discision horizontale du muscle sous-scapulaire (Figure 3). Ce qui rend l´emplacement de la butée difficile [6,13]. Toutefois la décision horizontale du sous-scapulaire est préférable de la ténotomie en L car cette dernière a un effet d'affaiblissement sur le sous-scapulaire [14]. La butée a été utilisée la majorité des cas en position debout et en quelque cas en position couchée. La comparaison des séries qui utilisent la butée en position debout comme Doursounian *et al*. [15] et Boileau *et al*. [16] ou couchée comme Hovelius *et al*. [13] ou Lafosse *et al*. [17] n´a pas montré une différence au terme de résultats cliniques. Également, la fixation de la butée a été effectuée par des vis 4,5 dans la majorité des cas et en quelque cas par des vis 3,5 mais cela n´a pas d´influence significative sur les résultats cliniques comme le montre les études biomécaniques de Willemot *et al*. [18].

L´arthrose gléno-humérale postopératoire est une complication fréquente dans les suites de l´intervention de Latarjet, son taux est très variable dans la littérature [5-9,12,19,20]. En effet, le recul moyen diffère énormément d'une série à l'autre, plus il est élevé, plus le taux d'arthrose postopératoire est important, et plus le stade de cette arthrose est avancé. Avec 13% d´omarthroses de stade 1 et 3% de stade 2, nous avons observé des taux d´omarthrose assez inférieure à ceux des séries de Singer *et al*. [9] 71% ou d´Allain *et al*. [21] 62%, celles-ci ayant respectivement 20 et 14 ans de recul. Par ailleurs Bouju *et al*. [20], avec un recul de 13 ans a constaté des taux plus faibles de 7,8%. Le taux faible d´omarthrose dans notre série peut être expliqué par le faible recul moyen qui était de 6,5 ans, en effet, le fait d'inclure dans la série des patients avec un recul de 2 et 3 ans tend à biaiser ce taux. Mais, comme d´autres auteurs [22], nous avons constaté qu´une butée sus équatoriale ou non affleurante était un facteur de risque majeur de développement d´une omarthrose.

## Conclusion

La technique de Latarjet dans le traitement de l´instabilité antérieure de l´épaule est une technique efficace avec un des bons résultats et un taux de satisfaction important chez les patients. Sa principale difficulté réside dans le bon positionnement de la butée coracoïdienne souvent gêné par l´exposition réduite qu´offre l´abord delto-pectorale. Ceci explique le taux important de mal positionnements de butées coracoïdiennes même par les chirurgiens les plus expérimentés. Avec les répercussions importantes de la position de la butée coracoïdienne sur la qualité des résultats cliniques nous soulignant l´intérêt de l´utilisation des outils qui pourrait améliorer le positionnement de la butée comme des guides de forage pour positionner la butée.

### État des connaissances sur le sujet

- L´instabilité antérieure de l´épaule est une pathologie qui atteint essentiellement des sujets jeunes et actifs;
- La technique de Latarjet constitue une des techniques les plus utilisées vu ses excellents résultats.

### Contribution de notre étude à la connaissance

- Le bon positionnement de la butée coracoïdienne au cours de la technique de Laterjet est souvent difficile même pour les équipes les plus expérimentées;
- La position de la butée coracoïdienne a un effet direct sur la stabilité et les douleurs de l´épaule en post opératoire;
- Une butée sus équatoriale ou non affleurante est un facteur de risque majeur de développement d´une omarthrose.
